# Synthesis, Surface Modification and Characterisation of Biocompatible Magnetic Iron Oxide Nanoparticles for Biomedical Applications

**DOI:** 10.3390/molecules18077533

**Published:** 2013-06-27

**Authors:** Mahnaz Mahdavi, Mansor Bin Ahmad, Md Jelas Haron, Farideh Namvar, Behzad Nadi, Mohamad Zaki Ab Rahman, Jamileh Amin

**Affiliations:** 1 Department of Chemistry, Faculty of Science, Universiti Putra Malaysia, 43400 UPM Serdang, Selangor, Malaysia; E-Mail: jaamin2000@yahoo.com; 2 Shiraz Branch, Islamic Azad University, Shiraz, 71993-3, Iran; 3 Centre of Foundation Studies for Agricultural Science, Universiti Putra Malaysia, 43400 UPM Serdang, Selangor, Malaysia; E-Mails: mdjelas@gmail.com (M.J.H.); mzaki53@gmail.com (M.Z.A.R.); 4 Institute of Tropical Forestry and Forest Products, Universiti Putra Malaysia, 43400 UPM Serdang, Selangor, Malaysia; E-Mail: farideh.namvar@gmail.com; 5 Mashhad Branch, Islamic Azad University, Mashhad, 917568, Iran; 6 Department of Civil and Structural Engineering, Faculty of Engineering and Built Environment, Universiti Kebangsaan Malaysia, 43600 UKM Bangi, Selangor, Malaysia; E-Mail: nadibehzad@gmail.com

**Keywords:** magnetic nanoparticles, surface modification, iron oxide

## Abstract

Superparamagnetic iron oxide nanoparticles (MNPs) with appropriate surface chemistry exhibit many interesting properties that can be exploited in a variety of biomedical applications such as magnetic resonance imaging contrast enhancement, tissue repair, hyperthermia, drug delivery and in cell separation. These applications required that the MNPs such as iron oxide Fe_3_O_4_ magnetic nanoparticles (Fe_3_O_4_ MNPs) having high magnetization values and particle size smaller than 100 nm. This paper reports the experimental detail for preparation of monodisperse oleic acid (OA)-coated Fe_3_O_4_ MNPs by chemical co-precipitation method to determine the optimum pH, initial temperature and stirring speed in order to obtain the MNPs with small particle size and size distribution that is needed for biomedical applications. The obtained nanoparticles were characterized by Fourier transform infrared spectroscopy (FTIR), transmission electron microscopy (TEM), scanning electron microscopy (SEM), energy dispersive X-ray fluorescence spectrometry (EDXRF), thermogravimetric analysis (TGA), X-ray powder diffraction (XRD), and vibrating sample magnetometer (VSM). The results show that the particle size as well as the magnetization of the MNPs was very much dependent on pH, initial temperature of Fe^2+^ and Fe^3+^ solutions and steering speed. The monodisperse Fe_3_O_4_ MNPs coated with oleic acid with size of 7.8 ± 1.9 nm were successfully prepared at optimum pH 11, initial temperature of 45 °C and at stirring rate of 800 rpm. FTIR and XRD data reveal that the oleic acid molecules were adsorbed on the magnetic nanoparticles by chemisorption. Analyses of TEM show the oleic acid provided the Fe_3_O_4_ particles with better dispersibility. The synthesized Fe_3_O_4_ nanoparticles exhibited superparamagnetic behavior and the saturation magnetization of the Fe_3_O_4_ nanoparticles increased with the particle size.

## 1. Introduction

Nanoscience is one of the most important research and development frontiers in modern science. Nanotechnology is now widely used throughout the pharmaceutical industry, medicine, electronics, robotics, and tissue engineering. The use of nanoparticle (NP) materials offers many advantages due to their unique size and physical properties [1]. Nanoparticles have been used to deliver drugs to target tissues and to increase stability against degradation by enzymes. The superparamagnetic nanoparticle is one of these nanoparticles, which can be manipulated by an external magnetic field to lead it to the target tissue [2]. Based on their unique mesoscopic physical, chemical, thermal, and mechanical properties, superparamagnetic nanoparticles offer a high potential for several biomedical applications, such as [3,4,5]: (a) cellular therapy such as cell labelling, targeting and as a tool for cell-biology research to separate and purify cell populations; (b) tissue repair; (c) magnetic field-guided carriers for localizing drugs or radioactive therapies; (d) magnetic resonance imaging (MRI); (e) tumor hyperthermia; (f) magnetofection. Furthermore, special surface coating of the magnetic particles require, which has to be not only non-toxic and biocompatible but also allow a targetable delivery with particle localization in a specific area. Magnetic nanoparticles can bind to drugs, proteins, enzymes, antibodies, or nucleotides and can be directed to an organ, tissue, or tumor using an external magnetic field or can be heated in alternating magnetic fields for use in hyperthermia. 

The release mechanism of drugs, the diffusion coefficient and the biodegradation rate are the main factors which govern the drug release rate. Here, drugs may be bound to the nanoparticles either within the production process of nanoparticles or by adsorption of drugs to nanoparticles [6]. Particles were injected into a certain part of the body and a magnet was placed close to the point of injection, such that the particles were retained at the location of the magnet. Moreover, by placing the magnet in the vicinity of some organs or extremities it was possible to increase the concentration of the drugs at that position. Another field of medical applications where magnetic nanoparticles are very useful is magnetic resonance imaging (MRI) [7]. Because of their tendency to accumulate with different density at different tissue compositions, one can use magnetic nanoparticles as contrast agents for the localization and diagnostics of brain tumors [8].

For biological and biomedical applications, magnetic iron oxide nanoparticles are the primary choice because of their biocompatibility, superparamagnetic behavior and chemical stability [9]. The nanostructure is based on an inorganic core of iron oxide, such as magnetite (Fe_3_O_4_), and maghemite (γ-Fe_2_O_3_), coated with a polymer such as dextran [10], chitosan [11], poly(ethylenimine) (PEI) [12], and poly(ethylene glycol) (PEG) [13].

Researchers have used implant magnetic nanoparticles in capillary tissue as seed to enhance the capture of magnetic drug carrier in the tissue [14]. Carriers can be prepared for poorly soluble drugs by grafting hydrophobic moieties such as deoxycholic acid and hydrophilic moieties such as glycidol on to chitosan [15]. From the fundamental scientific viewpoint, the synthesis of uniform-sized nanocrystals with controllable sizes is very important because the properties of these nanocrystals depend strongly on their dimensions and also all biomedical application requires that the nanoparticles have high magnetization values, with sizes smaller than 100 nm, and a narrow particle size distribution [16].

Methods for preparing iron oxide magnetic nanoparticles (Fe_3_O_4_ MNPs) include microemulsion, thermal decomposition, and co-precipitation method. As one convenient and cheap method, chemical coprecipitation has the potential to meet the increasing demand for the direct preparation of well dispersed (water-base) Fe_3_O_4_ nanoparticles. This method offer a low-temperature alternative to conventional powder synthesis techniques in the production of nanopaticles, and the sizes of nanopaticles can be well controlled by apt surfactant [9]. Chemical coprecipitation can produce fine, high-purity, stoichiometric particles of single and multicomponent metal oxides [17].

On the other hand, magnetic iron oxide NPs has a large surface-to volume ratio and therefore possesses high surface energies. Consequently, they tend to aggregate so as to minimize the surface energies. Moreover, the naked iron oxide NPs have high chemical activity, and are easily oxidized in air (especially magnetite), generally resulting in loss of magnetism and dispersibility. Therefore, it is important to provide proper surface coating and developing some effective protection strategies to keep the stability of magnetic iron oxide NPs. These strategies comprise grafting of or coating with organic molecules, including small organic molecules or surfactants, polymers, and biomolecules, or coating with an inorganic layer, such as silica, metal or nonmetal elementary substance, metal oxide or metal sulfide. Practically, it is worthy that in many cases the protecting shells not only stabilize the magnetic iron oxide NPs, but can also be used for further functionalization [18]. 

The magnetic structure of the surface layer usually is greatly different from that in the body of nanoparticle, and the magnetic interactions in the surface layer could have a notable effect on the magnetic properties of nanoparticles [19]. Therefore, the interaction between the surfactant and the nanoparticle is critical and essential to synthesis and application of nanoparticles.

Oleic acid (OA) is a commonly used surfactant to stabilize the magnetic nanoparticles with strong chemical bond between the carboxylic acid and the amorphous iron oxide nanoparticles [20]. For this report, we studied oleic acid as biological molecules for coating the surface of the Fe_3_O_4_ to control the particle size, to prevent the nanoparticles from aggregation, to achieve biocompatibility, to increase lipophilicity and stability [21]. Generally preparation of MNPs for biomedical applications involved three steps. Firstly, the preparation of magnetic nanoparticles of required size, secondly, the encapsulation of the nanoparticles using suitable polymer with active functional groups and lastly attachment of a desired functional group such as amine which is suitable to bind a targeted active component such as drug for medical applications. Although many papers regarding preparation of Fe_3_O_4_ MNPs using oleic acid as surfactant have been published [21], they usually lack of experimental details. In this paper we described the experimental detail for preparation of Fe_3_O_4_ MNPs coated with oleic acid as surfactant to determine the optimum pH, initial temperature and stirring speed in order to obtain the MNPs with small particle size and size distribution as well as high magnetization value that is needed for biomedical application. The MNPs were characterized by Fourier transform infrared (FTIR), scanning electron microscope (SEM) coupled with an energy dispersive X-ray detector (EDX), transmission electron microscopy (TEM), powder X-ray diffraction (XRD), vibrating sample magnetometry (VSM), and thermogravimetric analysis (TGA). The chemical structure and the amount of the surfactant attached on the magnetite nanoparticles were also discussed.

## 2. Results and Discussion

### 2.1. Mechanism of the Fe_3_O_4_ MNPs Formation

During the precipitation of Fe_3_O_4_ from Fe^2+^ and Fe^3+^ salts mixtures, two separate reactions could occur after addition of ammonium hydroxide to observe the precipitation of Fe_3_O_4_ MNPs. It was well known that Fe(OH)_2_ and Fe(OH)_3_ formed at pH > 8 by the hydroxylation of the ferrous and ferric ions under anaerobic conditions [22]. Consequently, the formation of Fe_3_O_4_ MNPs occurred with black precipitation. The possible reaction for the formation of Fe_3_O_4_ MNPs as follows:

Fe^3+^ + 3OH^−^ → Fe(OH)_3_

Fe(OH)_3_→ FeOOH + H_2_O

Fe^2+^+ 2OH^−^ → Fe(OH)_2_

2FeOOH + Fe(OH)_2_ → Fe_3_O_4_↓ + 2H_2_O

The reaction is fast, very high yielding, and magnetite crystals are seen instantaneously after addition of the iron source. It is essential the whole reaction mixture be free of oxygen, otherwise magnetite can be oxidised to ferric hydroxide (γ-Fe_2_O_3_) in the reaction medium. The larger particles sizes can also be obtained by aggregation of small crystallites through syndesis [23].

### 2.2. Optimized Conditions for Synthesis of Fe_3_O_4_ MNPs

#### 2.2.1. Effect of pH Variation on Particle Size

**Figure 1:**
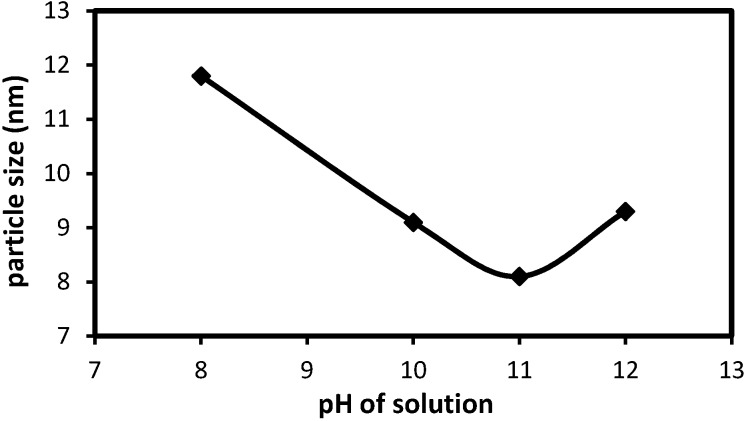
Effect of the solution pH on sizes of Fe_3_O_4_ MNPs (Temp: 45 °C, stirring rate: 800 rpm).

The pH range during the synthesis of iron oxide NPs should be 8-11 with maintaining molar ratio of Fe^3+^/Fe^2+^ (2:1) under a non oxidizing condition. The effect of pH on the average particle size of Fe_3_O_4_ MNPs as estimated from the Scherrer equation of the XRD peaks is presented in Figure 1. The figure shows the sizes of Fe_3_O_4_ MNPs reduce with the increase of solution pH when the pH is lower than 11, and also the particle size of Fe_3_O_4_ MNPs increase with the increase of solution pH when the pH is higher than 11. After increasing of the pH solution, the hydrolysis of Fe^3+^ occurred and Fe(OH)_3_ was generated in the first step. Then, Fe(OH)_2_ was generated as the pH of the reaction system increased, which was attributed to the hydrolysis of Fe^2+^. Finally, Fe_3_O_4_ can just be formed as the pH of the solution is further increased. This result shows that the growth of Fe_3_O_4_ nucleus occurred when the pH of solution is lower than 11, while the growth of Fe_3_O_4_ nucleus is easier to happen when the pH of solution is higher than 11 [24]. Therefore the pH of 11 will be selected for the optimum pH in further experiment.

#### 2.2.2. Effect of Temperature on Particle Size

**Figure 2:**
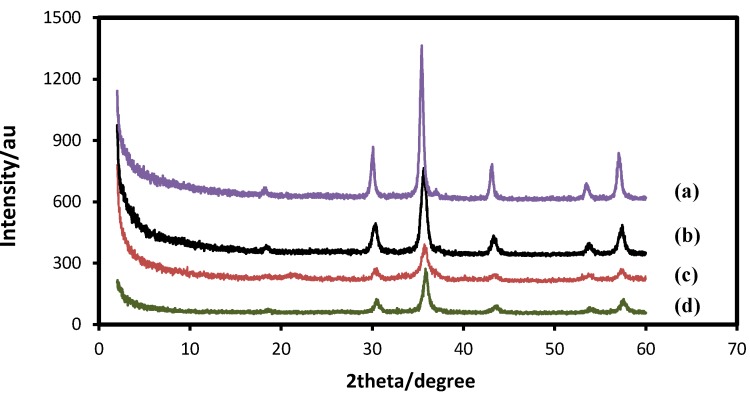
XRD diffraction patterns of the Fe_3_O_4_ MNPs at different initial temperatures; (**a**) 85 °C , (**b**) 65 °C, (**c**) 45 °C, and (**d**) 25 °C (pH: 11, stirring rate: 800 rpm).

The effect of temperature at the beginning of synthesis on particle size of Fe_3_O_4_ MNPs was investigated from 25 to 85 °C, and the XRD results are shown in Figure 2. The results indicated that all the nanoparticles were in spinel structure with face-centered cubic phase. The result showed that the intensity of the Bragg peaks increase by increasing the temperature. 

According to the estimated size from Scherrer equation, the crystallite sizes of Fe_3_O_4_ MNPs are reduced with the increase of reaction temperature from 25 to 45 °C and the crystallite size was increased from 8.3 nm to 13.2 nm when the temperature increased from 45 to 85 °C, implying there was greater polydispersity in reactions at higher temperatures. Increasing the reaction temperature would reduce the extent of aggregation of Fe_3_O_4_ nucleus and reduce sizes of Fe_3_O_4_ nanoparticles. However, the growth of Fe_3_O_4_ nucleus is easier to happen when the temperature is higher than 45 °C, resulting in larger size nanoparticles when the temperature is higher than 45 °C at the beginning synthesis of nanoparticles. On the other hand, a plausible explanation for this is that by increasing the reaction temperature there is more energy within the solution, this would increase mobility and cause a greater number of collisions between the particles [25]. Therefore, the initial temperature of 45 °C will be used for this experiment. 

#### 2.2.3. Effect of Stirring rate on Particle Size

**Figure 3:**
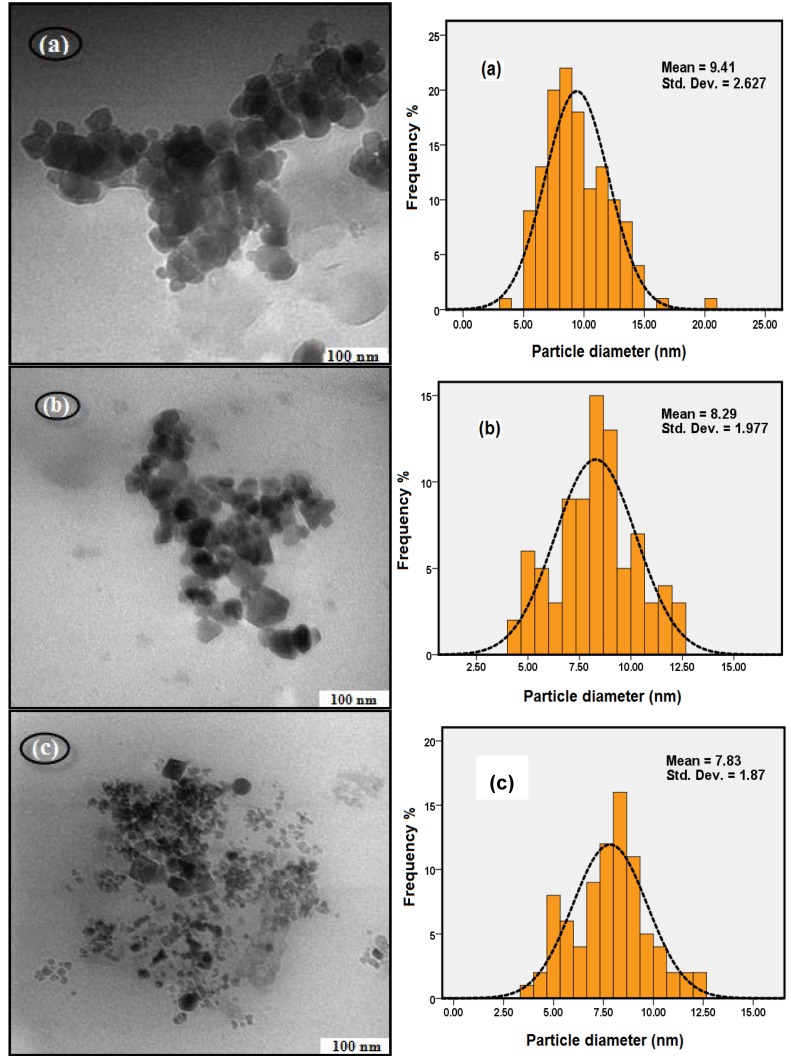
TEM images of Fe_3_O_4_ MNPs at different Stirring rate; (**a**) 400 rpm, (**b**) 600 rpm, and (**c**) 800 rpm (pH: 11, initial Temp: 45 °C).

The TEM results in Figure 3 show that when the stirring rate was increased from 400 rpm to 800 rpm, the average sizes of the Fe_3_O_4_ nanoparticles were decreased from 9.41 nm to 7.83 nm. When the stirring rate is increased, the energy transferred to the suspension medium is also increased and the reaction solution can be dispersed into smaller droplets and the size is reduced [24]. Another explanation for this reduction was the anomalous diffusion of particles at higher degree of agitation reduced the growth kinetics of the particles, and resulted in the smaller sized particles [26]. 

There is no change in the particle size of Fe_3_O_4_ MNPs, when the stirring speed is higher than 800 rpm. And from the experiment, we can see great deals of bubbles were generated with splashing of the reaction solution, when the stirring speed was higher than 800 rpm. Consequently, Fe_3_O_4_ MNPs will be easily oxided. Therefore 800 rpm was considered as the best stirring rate for this experiment.

### 2.3. Magnetic Properties of Magnetite NPs with Different Particle Size

**Figure 4:**
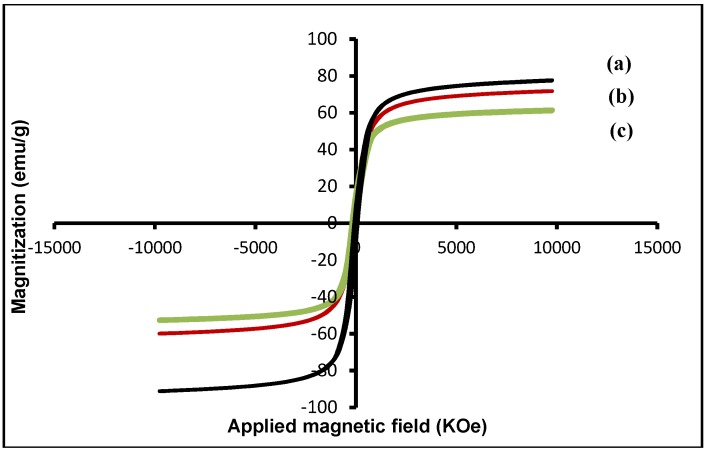
Magnetization curves obtained at different sizes of Fe_3_O_4_ MNPs; (**a**) 9.41, (**b**) 8.29, and (**c**) 7.83 nm.

Magnetic characterization of the magnetite NPs at different size is shown in Figure 4. It is clear, all the nanoparticles exhibit superparamagnetic behavior and have lower saturation magnetization values than the bulk Fe_3_O_4_ (~92 emu g^−1^) [27]. From Figure 4, the saturation magnetization (Ms) of the Fe_3_O_4_ MNPs increase from 58.60 to 78.00 emu g^−1^ with increase of the nanoparticle sizes from 7.83 to 9.41 nm. This result was due to the surface order/disorder interaction of the magnetic spin moment and the disturbance in the spinel structure inversion as a result of Laplace pressure [28] and also the increase of weight and volume of magnetite nanoparticles.

### 2.4. Characterization of Pristine and Oleic Acid Modified Fe_3_O_4_ MNPs

The properties of the pristine and oleic acid modified Fe_3_O_4_ MNPs prepared under optimum conditions were characterized and compared.

#### 2.4.1. Infrared Spectroscopy (FTIR)

**Figure 5:**
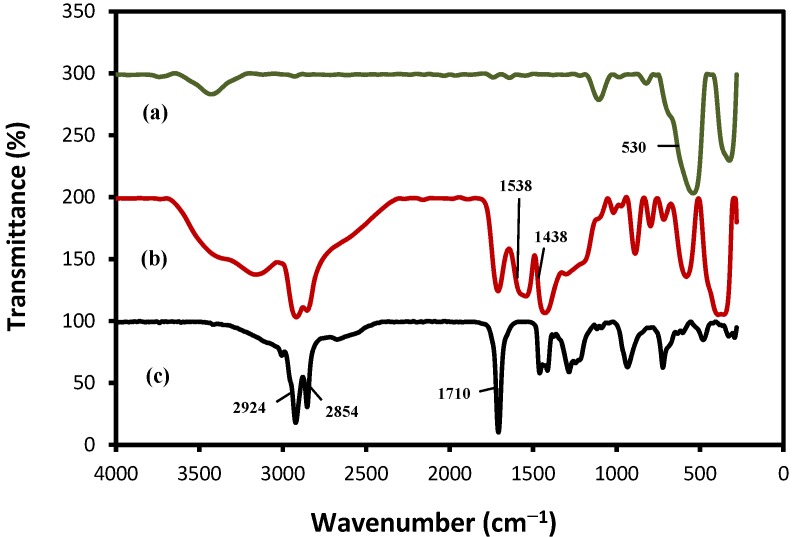
FTIR spectra of: (**a**) Fe_3_O_4_, (**b**) Fe_3_O_4_/OA, and (**c**) pure OA.

Figure 5 shows the FTIR spectra of the pristine Fe_3_O_4_ (a), Fe_3_O_4_ MNPs coated with OA (b), and pure oleic acid (c). For both sample (a) and (b), the analysis indicated absorption peaks at 530 cm^−^^1^ corresponding to the Fe–O vibration related to the magnetite phase [29]. In curve (c), two absorption peaks at 2,924 and 2,854 cm^−1^ were attributed to the asymmetric CH_2_ stretching and the symmetric CH_2_ stretching, respectively [30]. The intense peak at 1,710 cm^−1^ was due to the overlapping of the absorption bands of the carboxyl groups and the double bonds of OA. In the curve (b), the asymmetric CH_2_ and the symmetric CH_2_ stretching bands shifted to 2,921 and 2,850 cm^−1^, respectively. 

The results of the significant shift of these specific peaks to the lower frequency indicated that the hydrocarbon chains in the monolayer surrounding the nanoparticles were in a closed-packed, crystalline state [31]. OA-coated Fe_3_O_4_ MNPs infrared spectra indicated the presence of two bands, 1,438 cm^−1^ (*v*_s_: COO^–^) and 1,538 cm^−1^ (*v*_as_: COO^−^) (curve b), attributed to the oleate ion immobilized on the magnetite surface [32].

According to [33], and combined with other studies of carboxylates, the interaction between the carboxylate head and the metal atom was categorized as three structures:

• Structure I: unidentate complex where one metal ion is binding with one carboxylic oxygen atom

• Structure II: bidentate complex (chelating) where one metal ion is binding with two carboxylate oxygens

• Structure III: bridging complex where two metal ions are binding with two carboxylate oxygens

**Figure 6:**
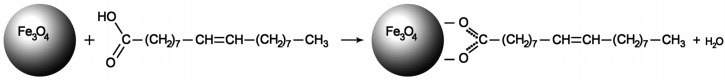
Chelating bidentate interaction between the COO^–^ group of oleic acid and the iron atom.

The wavenumber separation (Δ*v_o_*), between the *v*_as_(COO^–^) and *v*_s_(COO^–^) IR bands can be used to distinguish the type of the interaction between the carboxylate and the metal atom. The largest Δ*v_o_* (200–320 cm^−1^) was corresponding to the monodentate interaction and the smallest Δ*v_o_* (<110 cm^−1^) was for the chelating bidentate. The medium range Δ*v_o_* (140–190 cm^−1^) was for the bridging bidentate. In this work, the Δ*v_o_* value equal to 100 cm^−1^ (1,538 – 1,438 = 100 cm^−1^) indicate the existence of a bidentate structure II or bidentate chelation that two oxygen atoms of the carboxylic group are coordinated to the surface iron atoms [34]. This result demonstrated that the bonding pattern of the carboxylic acids on the surface of the nanoparticles was a combination of molecules bonded symmetrically and molecules bonded at an angle to the surface. From the above observation, we can confirm that OA was chemisorbed onto the Fe_3_O_4_ MNPs as a surfactant (Figure 6).

#### 2.4.2. X-Ray Diffraction Analysis (XRD)

**Figure 7:**
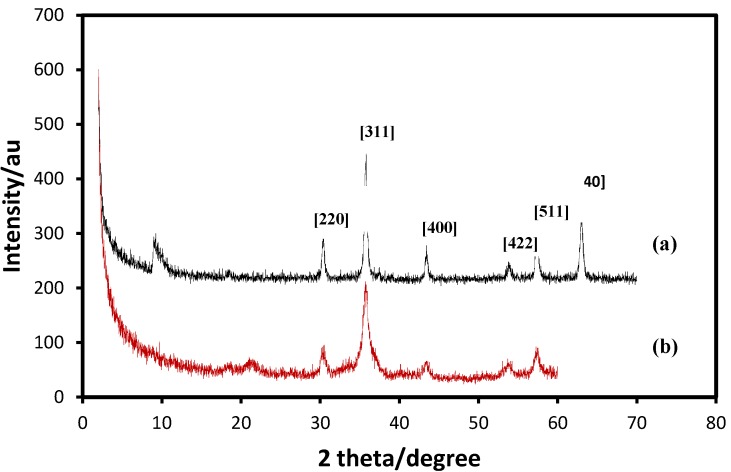
XRD patterns of (**a**) Fe_3_O_4_, and (**b**) Fe_3_O_4_/OA.

Figure 7 shows the XRD patterns of the pristine Fe_3_O_4_ (a), and Fe_3_O_4_ MNPs coated with OA (b). A series of characteristic peaks were observed in the XRD pattern at 2θ of 9.6°, 30.1°, 35.5°, 43.1°, 54.5°, 57.6° and 63.6° corresponding to the diffractions of 220°, 311°, 400°, 422°, 511° and 440° crystal faces of Fe_3_O_4_ spinel structure. The positions and relative intensities of the reflection peak of Fe_3_O_4_ MNPs agree with the XRD diffraction peaks of standard Fe_3_O_4_ samples [35], indicating that the black-colored magnetic powders are magnetite nanoparticles. Sharp peaks also suggest that the Fe_3_O_4_ nanoparticles have good crystallize structure. Peak broadening observed is consistent with the small particle size [36]. It was found that the magnetite crystallites could be well indexed to the inverse cubic spinel structure of Fe_3_O_4_. The XRD data further suggest that the effect of the modifiers on the crystal structure of core/shell samples is negligible. 

#### 2.4.3. Transmission Electron Microscopy (TEM)

**Figure 8:**
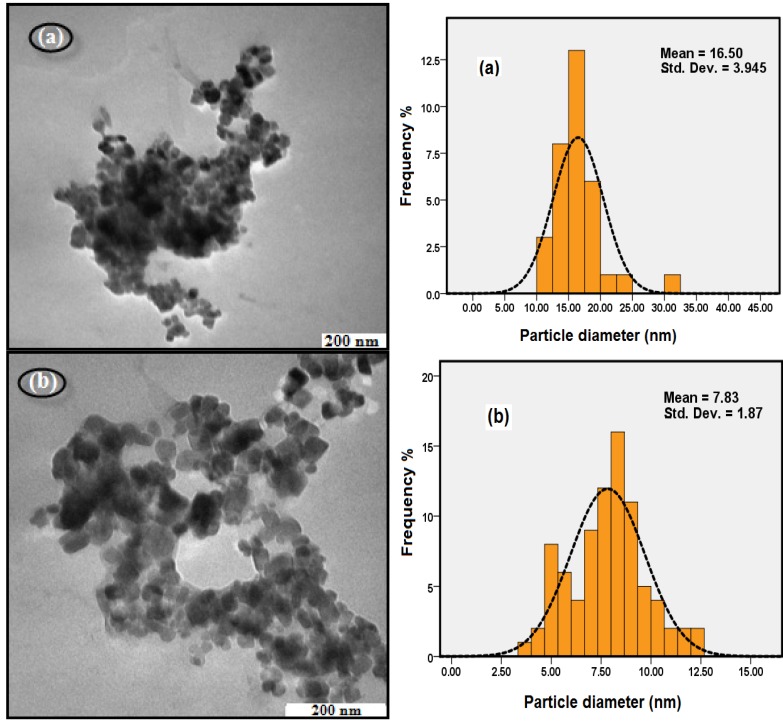
TEM images of (**a**) Fe_3_O_4_, and (**b**) Fe_3_O_4_/OA.

Figure 8 shows TEM images and size distribution of pristine Fe_3_O_4_ (a), and Fe_3_O_4_ MNPs coated with OA (b). It can be seen that the pristine Fe_3_O_4_ NPs were polydisperse and seriously aggregated. After surface modification by oleic acid the particles maintained their original cubic shape with a good monodispersity. The particle size is very uniform with the average size of about 7.83 nm. The average particle size of the pristine Fe_3_O_4_ MNPs was about 16.5 nm which was larger than the oleic acid modified Fe_3_O_4_ NPs. From the magnified image a slight aggregation can be observed which was due to the aggregation of individual particles with an incomplete coating by oleic acid molecules.

#### 2.4.4. Scanning Electron Micrograph (SEM)

**Figure 9:**
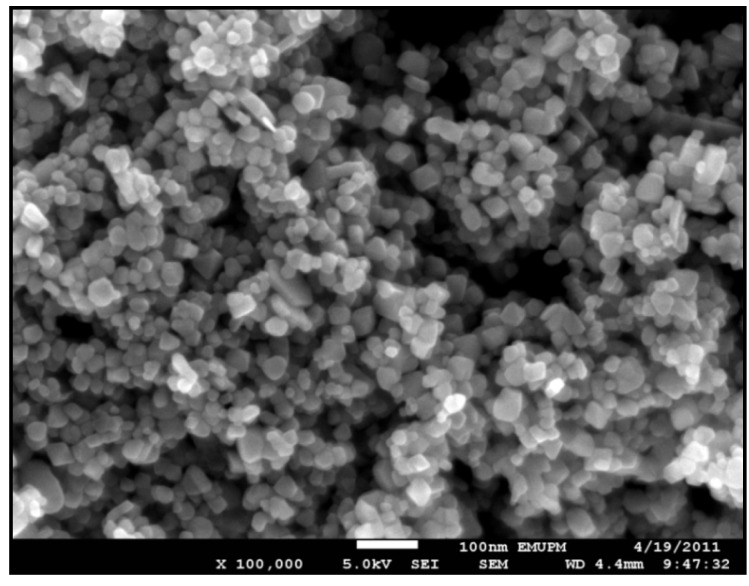
SEM micrograph of oleic acid modified Fe_3_O_4_ MNPs.

**Figure 10:**
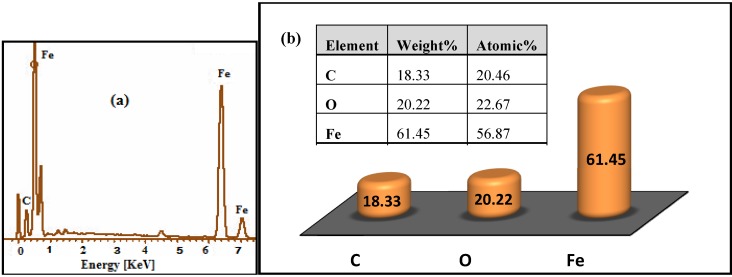
EDX spectra of (**a**) Fe_3_O_4_ MNPs, and (**b**) percentage of Fe, O, and C.

Figure 9 shows the SEM images for the iron oxide MNPs, which confirms that the Fe_3_O_4_ MNPs are cubic and highly uniform in size. In addition, Figure 10 shows the EDXRF spectra for the Fe_3_O_4_ MNPs coated with OA. As expected the peaks around 0.8, 6.3, and 6.8 keV are related to the binding energies of Fe. The spectrum contained three peaks, which were assigned to Fe, O, and C. The peak of C shows the existence OA functionality on the surface of iron oxide. The EDX analysis suggests that Fe, O and C (H could not be measured) are the main constituents in the magnetic NPs.

#### 2.4.5. Vibrating Sample Magnetometer (VSM)

**Figure 11:**
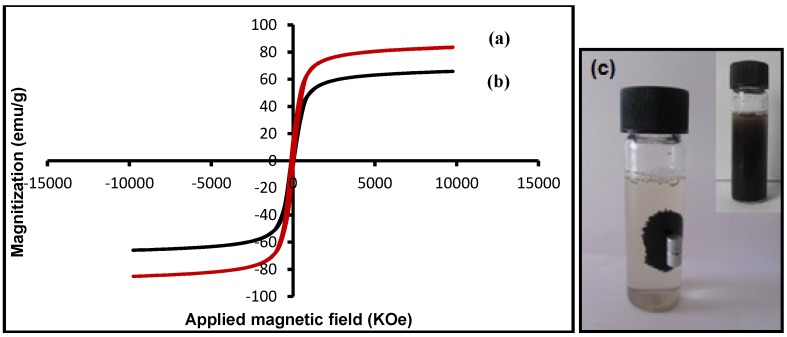
Magnetization curves of (**a**) Fe_3_O_4_, (**b**) Fe_3_O_4_/OA and (**c**) photograph of the separation of magnetic nanoparticles under an external magnetic field.

The magnetization curves measured at room temperature for pristine Fe_3_O_4_ and Fe_3_O_4_ MNPs coated with OA are compared in Figure 11. There was no hysteresis in the magnetization for both of samples, suggesting the magnetic particles produced are superparamagnetic. This can be attributed to the small size of NPs which were smaller than the superparamagnetic critical size (25 nm) [37]. On the other hand when the magnetic component size of the particles is smaller than critical size, the particles will exhibit superparamagnetism [38].

The saturation magnetization value was measured to be 81.40 emu g^−1^ for Fe_3_O_4_ and 58.60 emu g^−1^ for OA-coated Fe_3_O_4_. The high saturation magnetization of pure Fe_3_O_4_ indicated the good crystal structure. The saturation magnetization values of OA-coated Fe_3_O_4_ was smaller than the value for the pure magnetite nanoparticles, therefore the saturation magnetization was reduced after coating of oleic acid onto the surface of Fe_3_O_4_ MNPs. This was due to the existence of diamagnetic shell surrounding the magnetite nanoparticles which quench the magnetic moment [39]. However, both of them showed superparamagnetic behaviors, indicating that magnetite nanoparticles were incorporated in the composite particles, which exhibited no remanence effect from the hysteresis loops at applied magnetic field. 

Superparamagnetism, that is responsiveness to an applied magnetic field without permanent magnetization, is an especially important property therefore, these magnetic properties are critical in the applications of the biomedical and bioengineering fields. The magnetic response of Fe_3_O_4_ MNPs was tested by placing a magnet near the glass bottle. The black particles were attracted toward the magnet; therefore the Fe_3_O_4_ MNPs can be separated from the emulsion under an external magnetic field, as shown in Figure 11c.

#### 2.4.6. Thermo Gravimetric Analysis (TGA)

**Figure 12:**
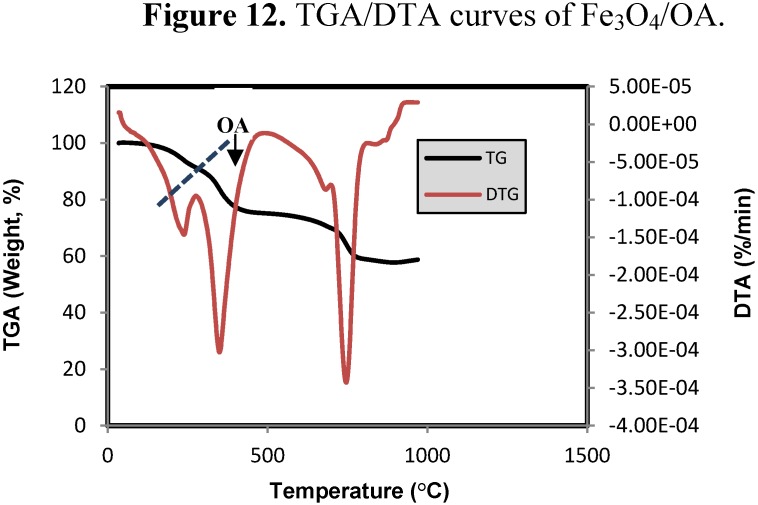
TGA/DTA curves of Fe_3_O_4_/OA.

The TGA/DTA curves of Fe_3_O_4_ MNPs coated with OA are shown in Figure 12. There are four derivative peaks in the DTA curve which related to the four mass losses in the TGA curve. 

The first peak is at about 242 °C, which is around the boiling or decomposition point of oleic acid, and the percentage of mass loss is about 19.8%, which possibly due to the removal of free oleic acid on the surface of Fe_3_O_4_ MNPs. The second peak is at 350 °C and the percentage of mass loss is about 22%, which confirms strong binding between the oleic acid molecules and Fe_3_O_4_ MNPs.

The third peak is at 682 °C, and the percentage of mass loss is about 2.5% which is attributed to the phase transition from Fe_3_O_4_ to FeO, because FeO is thermodynamically stable above 570 °C in phase diagram of the Fe-O system. The fourth derivative peak at 742 °C, related to a percentage mass loss of 14%, possibly because of the deoxidation of FeO since the TGA/DTA analysis was achieved under the N_2_ atmosphere [40]. Consequently from Figure 12 the content of surfactant was estimated to be about 18 wt %.

## 3. Experimental

### 3.1. Materials

Ferric chloride hexahydrate (FeCl_3_.6H_2_O, 97%) and ferrous chloride tetrahydrate (FeCl_2_.4H_2_O, 99%) were purchased from Merck (Darmstadt, Germany). Oleic acid (OA) was obtained from Sigma-Aldrich (St. Louis, MO, USA). Ethanol and ammonium hydroxide (25 wt%) were all used as supplied. Distilled water is also used for preparation of the solutions. All other chemicals were of analytical reagent grade and were used without further purification.

### 3.2. Synthesis of Modified Magnetite Nanoparticles

The magnetite nanoparticles were prepared by a co-precipitation method. FeCl_3_·6H_2_O (about 0.046 mol) and FeCl_2_·6H_2_O (about 0.023 mol) were dissolved in deionized water (150 mL) in a 250 mL three-necked flask and heated to the required temperature (85, 65, 45 and 25 °C). 

The solution was bubbled with nitrogen gas to prevent unwanted oxidation. Then ammonium hydroxide (20 mL, 25%) was added quickly in to the iron solution under vigorous stirring (800 rpm) until the pH value of the solution reached to required pH (8, 10 and 11). After 30 min oleic acid (OA, 3 mL) was added into the mixture to modify the Fe_3_O_4_ MNPs and the mixture was heated to 80 °C. After 1 h, the resulting Fe_3_O_4_ MNPs (black precipitate) were collected from the solution by magnetic separation and washed several times with deionized water and ethanol, then dried under vacuum conditions at 60 °C for 12 h.

### 3.3. Characterization Methods and Instruments

FT-IR spectra of the Fe_3_O_4_ MNPs were recorded over the range of 400–4,000 cm^−1^ by a model spectrum 100 series (Perkin Elmer, Walthman, MA, USA) FTIR spectrophotometer. The crystalline structure and phase purity of the Fe_3_O_4_ MNPs pro­duced were identified by X-ray diffraction measurement (XRD-6000; Shimadzu, Tokyo, Japan). The sizes of the nanoparticles were evaluated from the XRD data using the Debye-Scherrer equation, which gives a relationship between particle size and peak broadening by the following equation:

d = kλ/(β·cosθ)

where *d* is the particle size of the crystal, *k* is Sherrer constant (0.9), *λ* is the X-ray wavelength (0.15406 nm), *β* is the line broadening in radian obtained from the full width at half maximum, and *θ* is the Bragg diffraction angle of the XRD diffraction patterns. Magnetic properties of the samples were measured using a vibration sample magnetometer (VSM; Lake Shore Model 7400, Tokyo, Japan) under magnetic fields up to 10 kOe. The thermal properties were determined using the thermogravimetric analysis (Perkin−Elmer, Model TGA−7). Transmission electron microscopy (TEM) observations were carried out on a Hitachi H-7100 electron microscope (Hitachi, Tokyo, Japan) with an acceleration voltage of 200 kV and the particle-size distributions were determined using the UTHSCSA Image Tool version 3.00 program. Scanning electron microscopy (SEM) was performed using a Philips XL-30 instrument (Philips, Eindhoven, The Netherlands) to study the morphology of magnetic NPs. The energy dispersive X-ray fluorescence spectrometry (EDXRF) was carried out on a DX-700HS spectrometer (Shimadzu).

## 4. Conclusions

This study investigated the synthesis of superparamagnetic iron oxide nanoparticles prepared by a co-precipitation method, using OA as surfactant. This was for the purpose of achievement for biocompatibility and thermal stability for use in magnetic hyperthermia for biomedical applications. Fourier transforms infrared spectra and X-ray diffraction showed that the Fe_3_O_4_ MNPs were successfully coated by oleic acid. The results showed that the crystallite and average particle size of the Fe_3_O_4_ MNPs were dependent on pH, temperature and stirring speed. The saturation magnetization of the MNPs was proportional to the particle size.
